# Towards Network-Based Neurofeedback of Visual Areas to Enhance Visual Perception

**DOI:** 10.1007/s10548-025-01172-5

**Published:** 2026-01-22

**Authors:** Despina Zlatkova, Stephan M. Gerber, Branislav Savic, Tobias Nef, Adrian G. Guggisberg, Marija Ušćumlić

**Affiliations:** 1 Division of Neurorehabilitation, Department of Clinical Neurosciences, University Hospital of Geneva, Av. de Beau-Séjour 26, Geneva, 1211 Switzerland; 2 Universitäre Neurorehabilitation, Universitätsklinik für Neurologie, Inselspital, University Hospital of Berne, Bern, 3010 Switzerland; 3 Gerontechnology and Rehabilitation Group, ARTORG Center for Biomedical Engineering Research, University of Bern, Bern, 3012 Switzerland; 4 Department of Neurology, Inselspital, Bern, 3010 Switzerland

**Keywords:** Neurofeedback, Alpha-band, Functional connectivity, Visual perception, EEG

## Abstract

α-band-mediated brain network communication underlies visual processing and is disrupted in visual deficits. We aimed to develop and evaluate an EEG-based neurofeedback targeting α-band connectivity of source-reconstructed visual areas with the rest of the brain, to determine whether modulating this network enhances detection of low-contrast visual stimuli. In this randomized, active-controlled study, 28 participants received real-time auditory neurofeedback designed to increase global α-band connectivity between target regions and the rest of the brain. Feedback targeted interactions with the visual cortex (V1–V3) in the active group and with the frontal cortex in the control group. Each participant completed two neurofeedback sessions on separate days. Resting-state connectivity, visual, and attentional performance were assessed on the days preceding and following training. 50% of participants in the active group successfully increased α-band connectivity of the targeted visual cortex, whereas connectivity decreased in the control and non-responder groups. Improvements in stimulus detection were observed primarily in male participants and appeared to be influenced by additional factors, including baseline performance levels. Even brief network-based neurofeedback interventions can enhance α-band connectivity of visual areas.

**Supplementary Information:**

The online version contains supplementary material available at https://doi.org/10.1007/s10548-025-01172-5.

## Introduction

Visual perception is essential to our ability to interact with and adapt to the environment. About 28–57% of stroke survivors develop visual field deficits (Hepworth et al. 2016; Rowe et al. 2019) which reduces their independence and quality of life (de Haan et al. 2015; Rowe et al. 2013). With few available restitution therapies and limited effectiveness (Hazelton et al. 2019), it is essential to understand the neural mechanisms of visual perception to develop effective interventions for visual field deficits. Previous neurophysiological investigations of vision have consistently associated task-induced changes in EEG α-band rhythm (8–12 Hz) to visual performance. Changes in α‐band power (Dijk et al. 2008; Iemi et al. 2017; Thut et al. 2006), α‐band phase (Busch et al. 2009; Mathewson et al. 2009), and α‐band frequency (Samaha and Postle 2015) have all been linked to fluctuations in perceptual awareness and detection performance.

In addition to these task-related changes, resting-state α activity has been implicated in vision. A substantial body of literature shows that spontaneous resting-state α activity reflects large-scale brain communication and facilitates long-range network integration (Chapeton et al. 2019; Guggisberg et al. 2008). Moreover, network communication as quantified with functional connectivity (FC) has been shown to predict motor and cognitive behavioral performance in both healthy individuals and neurological patients (Dubovik et al. 2012, 2013; Manuel et al. 2018; Mottaz et al. 2024).

Importantly, α-band mediated network communication is also critically involved in visual processing, influencing perceptual performance and visual attention (Allaman et al. 2020; Palva and Palva 2007). Resting and task-related α-band dynamics offer key insights into the neural basis of visual perception. Allaman et al. (2020) demonstrated that, in addition to stronger task-induced α power decrease during seen stimuli, stronger resting-state α-band connectivity between visual cortex and the rest of the brain precedes seen stimuli and correlates with better detection performance. Importantly, participants with better overall visual detection performance showed stronger α resting-state FC of visual areas, while those with poorer performance exhibited stronger task-induced activations. These findings suggest that resting-state α-band connectivity may support perceptual efficiency, whereas task-induced activations act as compensatory mechanisms when spontaneous network interactions are weaker.

In patients with homonymous visual field deficits following brain injury, resting-state α-band FC between associative visual cortices and the rest of the brain is significantly reduced (Allaman et al. 2021). This reduction correlates linearly with impairment severity and predicts clinical outcome better than task-based measures. Based on these observations, we hypothesized that increasing α-band FC between the visual cortex and the rest of the brain will improve visual perception.

One way to achieve this is through neurofeedback training, in which a target brain signal is continuously monitored and translated into a simple external cue (e.g., a tone, light, or vibration). By attending this feedback and practicing over multiple sessions, participants learn to upregulate or downregulate the targeted neural activity through operant conditioning (Sitaram et al. 2017). Neurofeedback protocols that target α-band connectivity between motor brain regions and the rest of the brain were introduced by Mottaz et al. (2015), who demonstrated that both healthy participants and stroke patients could increase α-band connectivity and improve motor performance when provided with real-time visual feedback based on functional connectivity (Mottaz et al. 2015, 2018).

We adopted this methodological approach originally developed for motor areas and applied it to more posterior visual regions to investigate whether α-band connectivity could be enhanced in healthy participants. Targeting the visual cortex with neurofeedback poses a specific challenge in the choice of feedback modality. Using visual feedback risks directly activating the region of interest, which could confound with the attempted upregulation through neurofeedback. To minimize this risk, we employed nonvisual feedback modalities, auditory and tactile. Our objective was to assess whether short-term neurofeedback training could strengthen α-band connectivity between visual areas and the rest of the brain and whether such modulation would be associated with improved detection of low-contrast visual stimuli.

## Methods

This study included an exploratory phase, which evaluated and compared the neural effects of auditory and tactile feedback modalities on α-band functional connectivity with visual cortical areas (see Supplementary Materials A). Its purpose was to identify the feedback modality with potential to support neurofeedback learning without inducing confounding activation near the targeted region.

The main experiment then utilized the selected modality to investigate neurofeedback efficacy under selected feedback conditions. The aim was to assess whether two days of the selected auditory neurofeedback training can enhance α-band connectivity in visual areas and improve visual perception, and whether these effects are region-specific. Throughout the manuscript, we analyze the α-band connectivity of a region of interest (ROI) with the rest of the brain. This measure, commonly referred to in the literature as ‘global connectivity,’ is hereafter termed ‘global α-band connectivity’ with the ROI for clarity and consistency.

### Participants

Twenty-eight neurotypical participants were randomly assigned, in a single-blind design, to either the active or control group (*n* = 14 per group). At study planning, only single-arm pilot data (Mottaz et al. 2015) were available. Based on a one-sample t-test against zero, the pilot mean (0.29, SD = 0.38) corresponded to a standardized effect size of Cohen’s d ≈ 0.76, indicating that at least 13 participants would provide ~ 80% power at a one-tailed α = 0.05. The primary analysis of the present study, however, involves an independent two-sample, one-tailed comparison of the FC slopes in the visual area (active > control). With the available sample size, this design achieves > 80% power only for large effect sizes (Cohen’s d ≳ 1). This limitation is acknowledged, and results are interpreted primarily in terms of effect estimates and confidence intervals rather than post-hoc power. Power analyses were performed using G*Power 3.1.9.

In the active group, there were 8 males and 6 females (mean age 25 ± 5.1 years), including one left-handed participant. The control group included 5 males and 9 females (mean age 26.86 ± 9.48 years) and two left-handed participants. All subjects had normal or corrected-to-normal vision, no history of neurological or psychiatric disorders, and no regular use of drugs or alcohol. Written informed consent was obtained from all participants, and the study was approved by the Ethics Committee of the Canton of Bern, in accordance with the Declaration of Helsinki. Participants received monetary compensation for their participation. The study was registered on ClinicalTrials.gov (NCT05732649, https://clinicaltrials.gov/study/NCT05732649). Here, we report Experiment 1 from this two-part investigation.

### Experimental Design

**Fig. 1:**
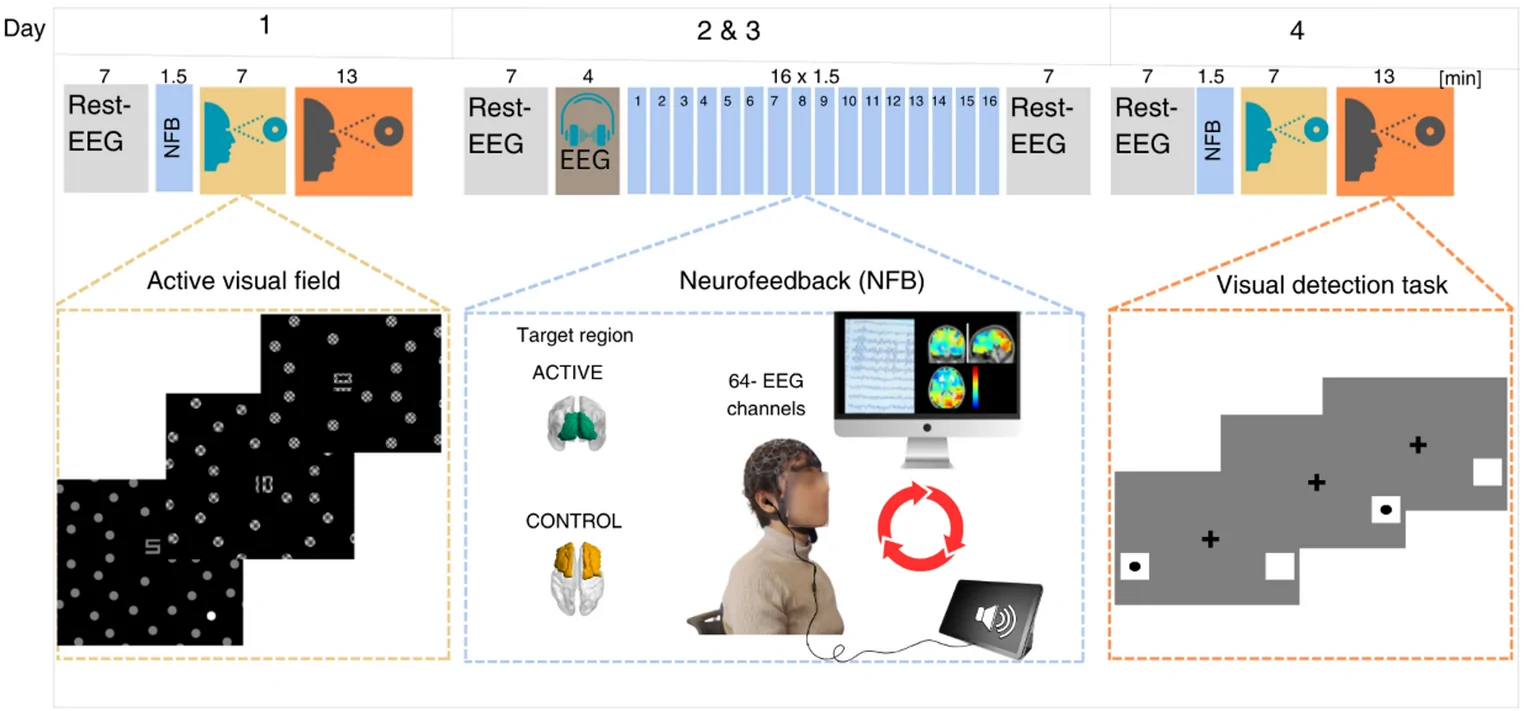
Experimental paradigm and timeline. On Days 1 and 4, participants underwent resting-state EEG, completed a short neurofeedback familiarization trial, and performed two visual assessment tasks. Days 2 and 3 consisted of resting-state EEG, passive auditory stimulation, and neurofeedback training in 16 blocks of 1.5 min each. In the neurofeedback box are shown the target regions for the active and control groups

The experiment consisted of four sessions (Fig. 1) conducted on separate days, with Sessions 1 and 4 dedicated to visual assessments and Sessions 2 and 3 to neurofeedback (NFB) training. Sessions 1 and 2, as well as Sessions 3 and 4, were performed on consecutive days, with a mean interval of 2.1 days (SD = 2.23) between the second and third sessions. Assessment sessions (Days 1 and 4) included a 7-minute eyes-closed resting-state EEG recording, a short NFB familiarization block (1.5-minute) and tests of visual perception and visual attention performance (see Sect. 2.3). NFB training sessions (Days 2 and 3) each began with a 7-minute eyes-closed resting-state EEG recording, followed by a four-minute period of passive auditory stimulation during which participants sat with their eyes closed and listened to continuous white Gaussian noise. Neurofeedback training then began, with a duration of 24 min divided into 16 blocks of 1.5 min each. The sessions ended with a final resting-state EEG recording.

Meditation practice was assessed with a binary yes/no question given evidence that experienced meditators perform better in real-time neurofeedback tasks (Garrison et al. 2013). Motivation was measured with two 5-point Likert scales evaluating the importance of monetary compensation and interest in neurofeedback (1 = very important, 5 = very unimportant). The subjective sense of control over the neurofeedback signal was rated after each session on a 5-point Likert scale (1 = no control, 5 = complete control).

### Visual Assessments

The assessment tasks were presented on an HP EliteDisplay E243 monitor (23.8 inches, 60 Hz refresh rate), with subjects seated at 54 cm from the screen.

*Visual Perception*. The visual detection task assessed participants’ ability to detect low-contrast stimuli. Stimuli were presented at six fixed contrast levels, with each level shown 24 times in a randomized sequence. Half of the stimuli appeared in the left visual field and the other half in the right visual field, plus 24 zero-contrast “catch” trials with no stimulus displayed, for a total of 168 randomized trials. A fixation cross (0.5° visual angle) was presented on a dark-gray background and two light‐gray squares (3 × 3°) positioned at ± 24° horizontally and 4° below fixation. Targets were low‐contrast circular Gaussian patches (0.5° visual angle) presented for 50 ms at the center of one of the square markers. Following stimulus offset, the cross and squares remained on screen for another 1.5 s (post-stimulus interval) to allow response collection. The fixation cross was refreshed at the start of each trial to signal onset.

Participants were instructed to maintain fixation on the central cross throughout the task and to respond only when confident that a target was present. They indicated the perceived location by pressing the left or right arrow key, corresponding to the side of the stimulus presentation. To estimate the contrast detection threshold, the proportion of correct responses was calculated at each contrast level. These proportions were used as input to fit a Weibull psychometric function. Due to limited contrast levels, noisy jittered estimates were added to the data, and the two-parameter Weibull function was fitted 100 times with varied initial parameters; the resulting threshold estimates were averaged to improve robustness and used as the main behavioral outcome. Thresholds were estimated separately for the left and right sides and then averaged, resulting in one contrast detection threshold per participant.

*Visual Attention.* To further assess changes in visual attention, we administered the Active Visual Field subtest from the TAP-M (Test of Attentional Performance – Mobility Version) (Zimmermann and Fimm 2021). In this standardized task, participants were required to detect color changes in peripheral gray circles while maintaining attention on a central task designed to discourage eye movements. Performance on this subtest reflects the functional extent of the visual field under divided attention. Further details on the test are available at https://www.psytest.net/en/test-batteries/tap-m/subtests#active_visual_field. We used the mean reaction time to the peripheral stimuli across all valid trials as a secondary behavioral outcome.

### EEG Acquisition and Pre-processing

EEG was sampled at 512 Hz using a 64-channel EEG cap (waveguard™ net, ANT Neuro) and referenced to the Cz electrode. The sponge electrodes in the silicone net cap were soaked in a potassium-chloride (KCl) saline solution prior to recording. Data were streamed and recorded using the Lab Streaming Layer (Kothe 2014).

For both the real-time (online) and the offline analysis, the EEG data were bandpass filtered between 1 and 20 Hz using elliptic filters (high-pass order = 5, low-pass order = 3), segmented into 1 s epoch and tapered with a Hanning window prior to Fourier transform.

For resting-state EEG recordings, participants were instructed to relax, minimize movement, and keep their eyes closed throughout the 7-minute recording period. No specific cognitive task was given, and participants were asked to remain awake and as still as possible.

### Source Localization and Functional Connectivity

The head model was based on the Montreal Neurological Institute (MNI) template, with solution points spaced 10 mm apart and constrained to gray matter regions. We computed forward models (lead fields) using a boundary element model from the Helsinki BEM library (Stenroos et al. 2007). An adaptive spatial filter (a scalar minimum-variance beamformer; Sekihara et al. 2004) was applied to the sensor recordings to reconstruct source-level time series. The beamformer weight vector was computed from the forward lead-field matrix and the covariance matrix of the recorded data. Dipole orientations were fixed such that they yielded maximum output signal-to-noise ratio at each solution point (Guggisberg et al. 2011; Sekihara et al. 2004). Henceforth, we use the term “voxel” for source reconstructions at solution points.

Imaginary coherence (IC) was calculated in the 8–12 Hz frequency band as our measure of functional connectivity (Nolte et al. 2004). For each voxel, weighted node degree (WND) was computed as the sum of IC values between that voxel and all other voxels, reflecting its global connectivity and relative importance within the brain network (Newman 2004; Stam and van Straaten 2012). To control for signal-to-noise variability, WND values were standardized via z-score normalization. Accordingly, throughout the paper, we refer to our main measure as “α-band FC” for clarity, though the more accurate term is “α-band WND”. All analyses were performed in MATLAB (The MathWorks, Version R2024b, RRID: SCR_001622) using NUTMEG (Dalal et al. 2011) and its Functional Mapping toolbox (Guggisberg et al. 2011). All visualizations were generated using custom MATLAB scripts, and brain network visualizations were created with BrainNet Viewer (Xia et al., 2013).

### Neurofeedback

Participants completed auditory neurofeedback training, with each group targeting global α-band FC of specific predefined brain ROI. The active group targeted the dorsal V3 (V3d) region, involved in lower visual field processing (Allaman et al. 2020). V3 neurons have low contrast detection thresholds (Eickhoff et al. 2005; Gegenfurtner et al. 1997; Kujovic et al. 2013; Larsson and Heeger 2006) and dorsal areas offer better EEG accessibility, making V3d a suitable neurofeedback target. Due to the limited spatial resolution of 64-channel EEG, combined with the use of a template MRI and precomputed spatial filters, the small V3d ROI was expanded to account for reduced localization precision. The neighboring visual areas V1, V2 and ventral V3 (V3v) from the Julich Brain Atlas (Amunts et al. 2020) were included to ensure broader coverage of the network of interest. The control group targeted the superior and middle frontal gyri, as defined by the Automated Anatomical Labeling (AAL) atlas (Tzourio-Mazoyer et al. 2002). For most participants from the control group, the ROIs were in the right hemisphere, except for the two left-handed participants, where the frontal cortex in the left hemisphere was targeted. The neurofeedback signal was translated into auditory feedback, where the volume of a white Gaussian noise reflected the strength of α-band FC between the target ROI and the rest of the cortex. Participants were instructed to try to increase the loudness of the sound by engaging in a mental strategy of their choice. They were encouraged to explore different mental states, including visualization, relaxation, or focused attention, and to identify and maintain the strategy that was most effective for them during training. The mental strategies reported by participants after each session are provided in Supplementary Information B (see Fig. S6).

Noisy channels were excluded from real-time analysis based on visual inspection. An adaptive spatial filter was precomputed using EEG data from passive auditory stimulation. During real-time analysis, data were processed in 300-ms steps using a sliding window of 500 ms, and the precomputed spatial filter was applied to the data segment. To balance timeliness and stability, α-band WND of voxels within the target ROI was estimated from the last 12 overlapping segments (the most recent ~ 3.8 s) using absolute imaginary coherence (Mottaz et al. 2015). To reduce the influence of possible outliers due to noise, the estimated value was normalized using a modified z-score (Iglewicz and Hoaglin 1993). 

Participants received neurofeedback through auditory stimuli, where the loudness reflected FC of the corresponding ROI in real time. Specifically, α-band WND values, reflecting the global FC with the target ROI, were translated into one of five discrete feedback levels using a stepwise mapping function. This function was tailored to the distribution of ROI z-scores obtained during passive stimulation. All real-time FC estimates of the target ROI were stored. Real-time FC values of two participants (one active, one control) had to be excluded, as their median FC showed extreme, unexplained jumps likely due to artifacts rather than physiological changes.

Neurofeedback effectiveness was defined as an increase in α-band FC over time in the targeted ROI and it was assessed in three complementary ways: (i) analyses of real-time FC modulation during NFB training, reflecting the dynamic, real-time signal that guided participants’ experience; (ii) offline NFB FC analyses included manual artifact rejection, spatial filters computed from the actual training data and FC estimated over larger blocks of epochs; this served as the primary and more reliable measure of training effects; and (iii) Resting-state FC analyses to determine whether effects carried over beyond the task.

For the real-time NFB FC analysis, saved α-band FC estimates from the targeted regions were summarized by calculating the median per block , and linear regression slopes (least-squares method) were fitted across both days combined (32 blocks ) for each participant. Consistent with early-stage training, within-day FC changes were variable and did not steadily increase, resulting in small and noisy slopes for individual days. Therefore, we report the pooled slope as a measure of overall learning.

### Offline FC Analyses

Neurofeedback and resting-state EEG recordings were visually inspected offline, and artifacts such as eye movements, muscle contractions, and electrode disturbances were excluded.

*For the neurofeedback data*, blocks from each training session were grouped into three 7.5-minute neurofeedback blocks. After preprocessing, we selected 300 artifact-free epochs from each block. The adaptive spatial filter was computed from epochs pooled across all three blocks and then applied to each neurofeedback block.

Analyses of *resting-state data* were performed from six assessments collected across four days. For each resting‑state block, we selected 300 artefact-free epochs and computed its spatial filter independently.

To evaluate neurofeedback effects in the active group, we focused on global FC changes of our primary ROI, dorsal V3 (Allaman et al. 2020), also analyzing left and right hemispheres separately to assess potential lateralized effects. For the control group, we analyzed changes in global α-band FC of their target region (frontal cortex). For most participants, this was in the right hemisphere, except for the two left-handed subjects, whose target was in the left frontal cortex.

Global α-band FC values were extracted at each ROI and time block, and for each subject, the slope was estimated by fitting a least-squares regression across the six-time blocks. We report the pooled slope across experimental Sessions 2 and 3 as the primary measure of overall learning.

### Responder Analysis

Classification of active-group participants into responders and non-responders was based on FC slope values (Kober et al. 2024) for dorsal V3, obtained from the offline neurofeedback analyses. We defined a data-driven threshold as the upper bound of the control group’s 99% confidence interval of slopes, with the additional requirement that slopes be positive. Slope values were compared between responders, non-responders, and controls. We compared baseline demographic, behavioral, and neurophysiological measures across the three groups.

To determine the neural characteristics of responders, we performed an exploratory seed analysis to examine baseline differences in connectivity between dorsal V3 and intraparietal sulcus regions (IPS; combined areas hIP1 and hIP2, Choi et al. 2006). This is motivated by the importance of occipito-parietal connections for visuo-spatial attention (Beffara et al. 2022).

### Statistical Analysis

To confirm group comparability at baseline, age was compared using an independent-samples t-test, motivation ratings and the subjective experience of controlling the neurofeedback signal were assessed with the Mann–Whitney U test, and differences in sex distribution, handedness, meditation practice, and corrected vision were evaluated with Fisher’s exact test.

In the analysis of real-time FC during NFB training, slopes of global α-band FC of target ROIs were tested against zero using a one-tailed t-test (slope > 0).

In the offline analysis, between-group differences in FC slopes across neurofeedback and rest time blocks, were assessed using one-tailed two-sample t-tests (active > control for the visual ROI; control > active for the control ROI). Within-group comparisons against zero were performed for all three ROIs. Bonferroni correction was applied to adjust for multiple comparisons in both the between-group and within-group analyses.

Model fit quality (R²) was summarized using median and IQR.

To check for spatial specificity of these FC changes, we additionally computed the global α-band FC slope at each voxel. Left-handed control participants were flipped along the midsagittal plane. Statistical significance was assessed using SnPM permutation testing (Singh et al. 2003) with 10,000 random permutations of the data. Because we aimed to evaluate whether any effects occurred outside the a priori ROI, we used uncorrected voxel-wise maps. Correcting for multiple comparisons would mask existing but weaker effects, making it impossible to assess spatial specificity.

Baseline differences across the responder, non-responder, and control groups were assessed using the following procedures. Continuous measures, including age, performance on the visual detection task, peripheral reaction times on the visual attention task, and baseline resting α-band FC, were analyzed using Kruskal–Wallis tests with Bonferroni corrected post hoc comparisons using the multiple-comparison test implemented in MATLAB’s multcompare function. Categorical measures, including sex, handedness, corrected vision, and meditation practice, were analyzed using Chi-square tests.

Slope values were compared between responders, non-responders, and controls using the Kruskal-Wallis test. Where significant differences were detected, post-hoc pairwise comparisons were applied with Bonferroni correction for multiple comparisons.

Behavioral data were analyzed using a mixed-model ANOVA with group (active vs. control) as a between‑subject factor and time (pre vs. post) as a within‑subject factor. In exploratory analyses, sex was also included. Main effects and interactions were tested, and post‑hoc comparisons were conducted using unpaired *t*-tests where appropriate with Bonferroni correction.

To further explore ceiling effects, we grouped contrast threshold data according to baseline performance using a median split on Day 1 thresholds. A two-way mixed ANOVA was then conducted with group (poor vs. good baseline) as a between-subjects factor and time (Day 1, Day 4) as a within-subjects factor.

Finally, a Spearman correlation was used to test whether slope values were associated with behavioral improvements in contrast threshold.

## Results

No baseline differences were detected between the active and control groups in age (t_(26)_ = − 0.65, *p* = 0.524), sex (Fisher’s exact, *p* = 0.449), handedness (Fisher’s exact, *p* = 1.000), corrected vision (Fisher’s exact, *p* = 0.252), or meditation practice (Fisher’s exact, *p* = 1.000). The groups also did not differ in the perceived importance of monetary compensation (Mann–Whitney: z = − 1.10, *p* = 0.271) or in interest in experiencing neurofeedback (Mann–Whitney: z < 0.001, *p* = 1.000). The subjective experience of controlling the neurofeedback signal, averaged across Sessions 2 and 3, was similar between groups (z = 0.53, *p* = 0.593), indicating successful blinding of the participants.

### Neurophysiological Effects

**Fig. 2:**
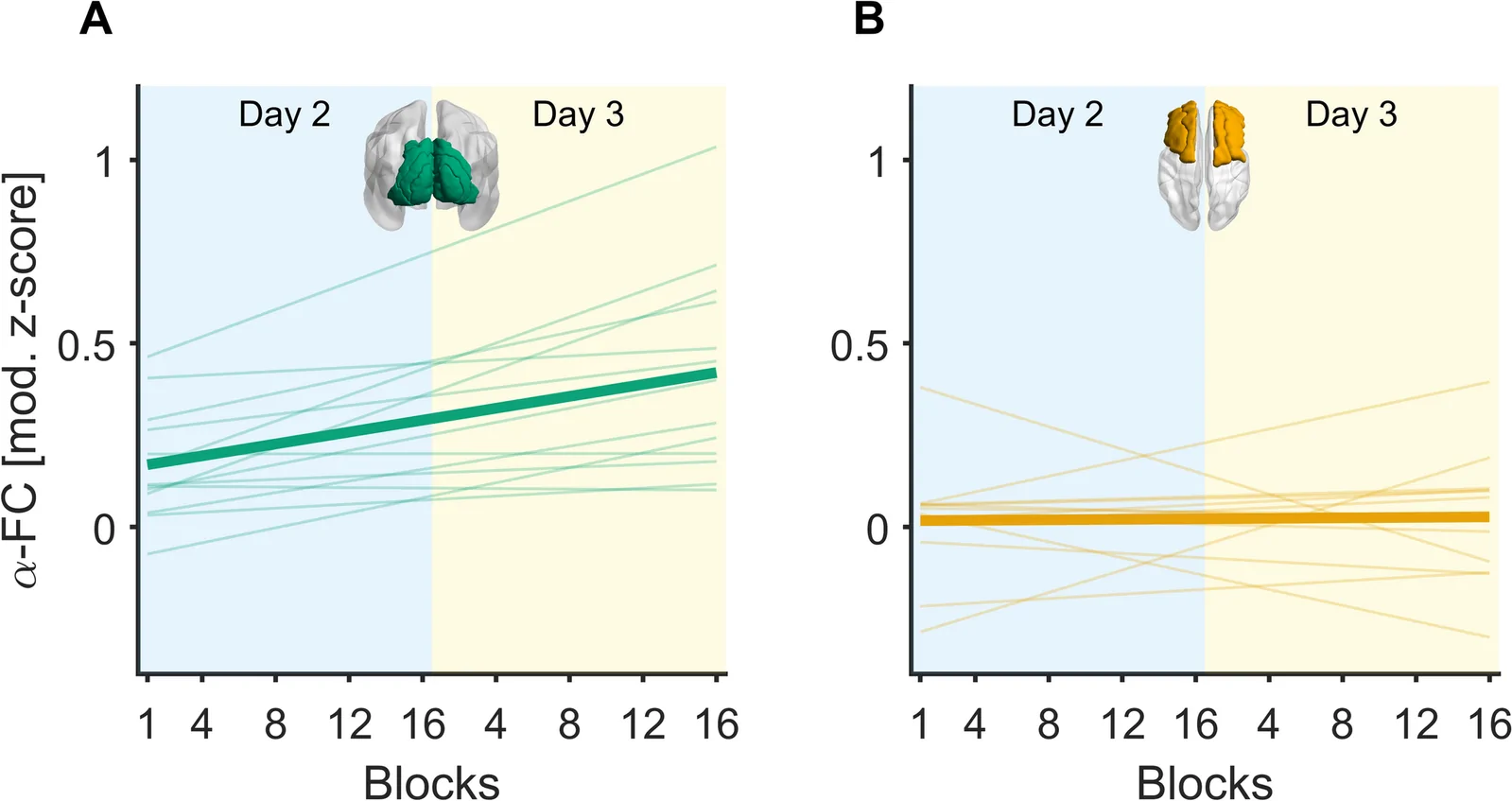
Neurofeedback online analysis. Fitted slopes of the real-time α-band FC for the active (**A**, green) and control (**B**, yellow) groups across two training days. Thin lines represent individual participants, and thick lines show the group mean slope. Background shading codes for protocol day 2 (blue) and day 3 (yellow)

Analysis of real-time neurofeedback FC values showed a significantly positive slope in the active group, reflecting an overall increase of global α-band FC of visual areas over the two-day NFB training (t(12) = 4.34, *p* < 0.001, 95% CI [0.148, ∞), d = 1.20). In contrast, the control group showed no significant increase (t(12) = 0.15, *p* = 0.441, 95% CI [–0.110, ∞), d = 0.04). Fitted slopes for both groups are shown in Fig. 2.

Group-level median FC values for each training block are shown for both groups across training days in Supplementary results B (Fig. S3).

**Fig. 3:**
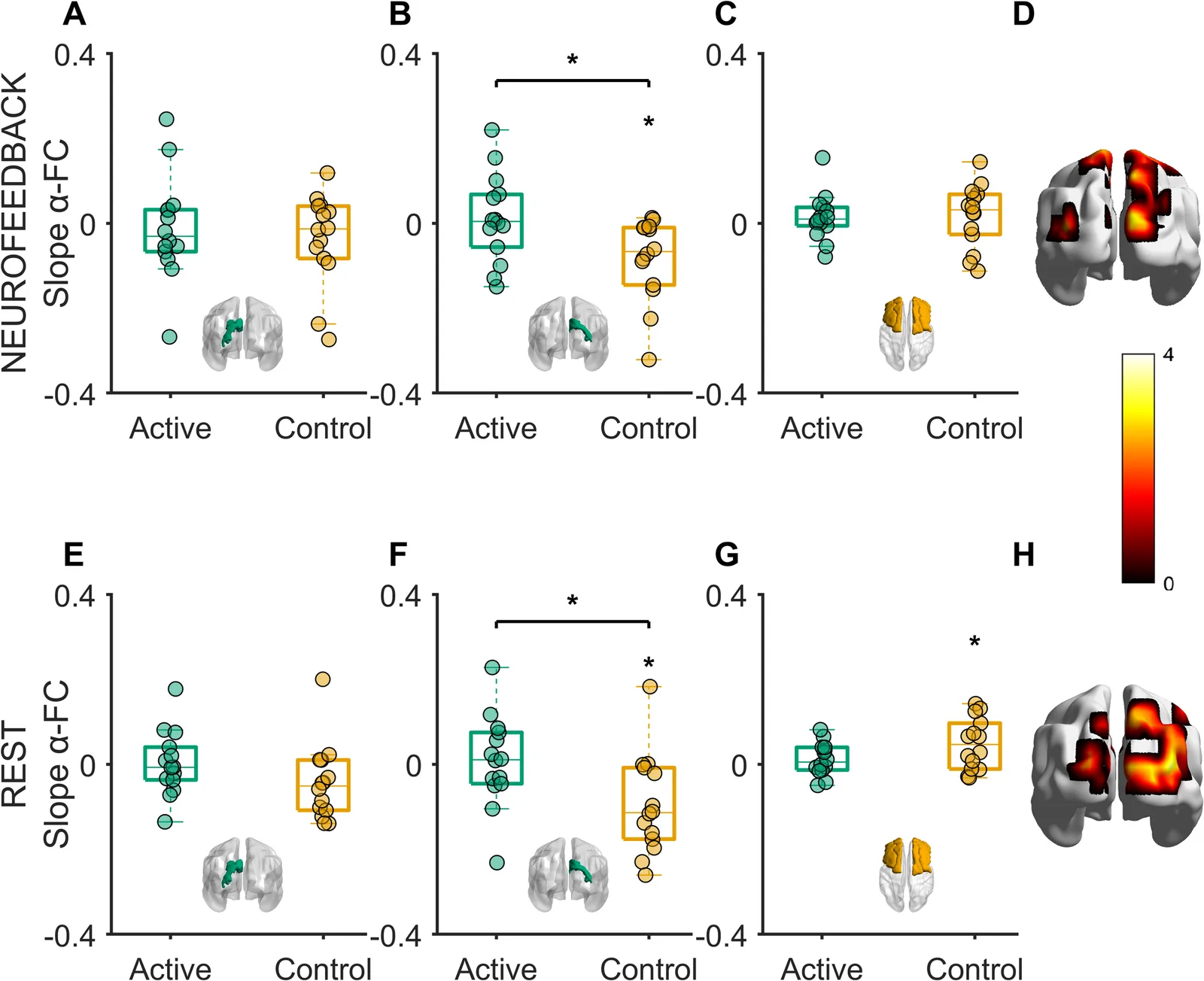
Neurofeedback and rest offline analysis. Slopes characterizing global α-band FC trends of the ROI shown on the 3D brain inlay for the active (green) and control (yellow) groups during neurofeedback training (**A–C**) and rest sessions (**E–G**). Individual participants are plotted as dots, and boxplots indicate the median, interquartile range. (**D**,** H**) Brain surface rendering illustrating voxels where the active group showed a greater increase in α-FC slope during neurofeedback and rest sessions compared with the control group (voxel-wise analysis, *p* < 0.05, uncorrected)

**Fig. 4:**
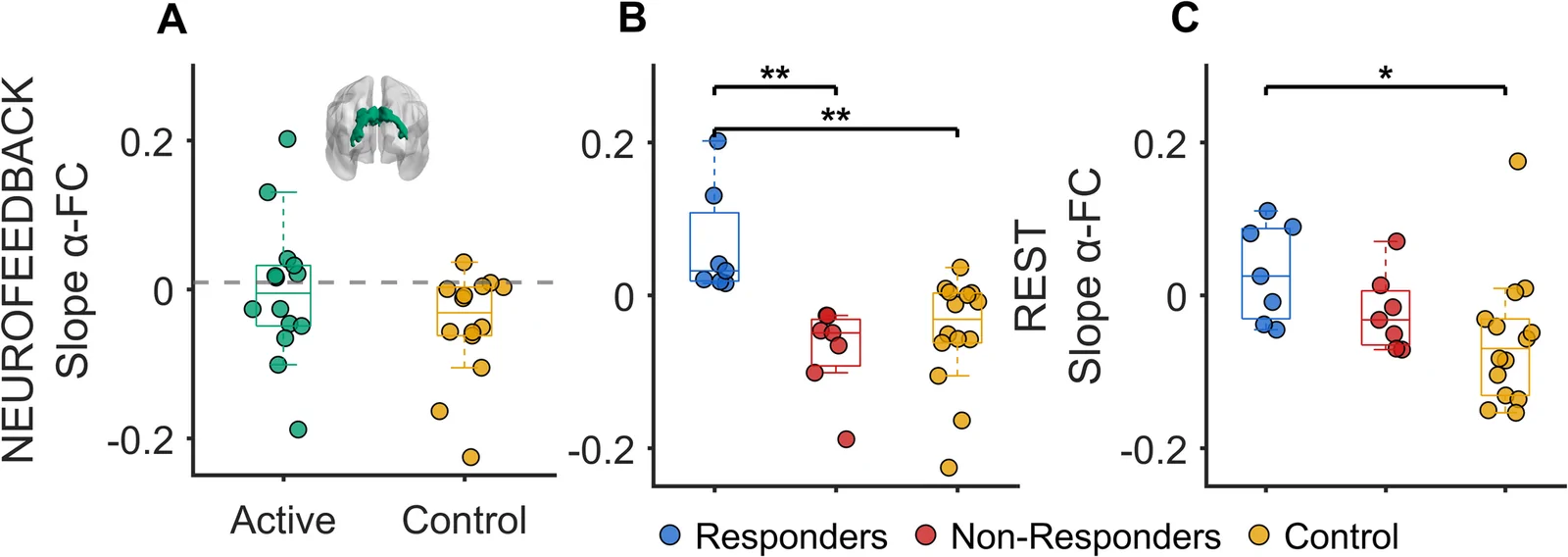
Classification of NFB responders and non-responders. (**A**) Individual slopes of α-band functional connectivity (α-FC) across the six neurofeedback training blocks for active and control groups in dorsal V3. The dashed line indicates the 99th CI of the control distribution, used as the cutoff to define responders (active group participants exceeding this threshold). (**B**) α-FC slopes of dorsal V3 during NFB for responders, non-responders, and controls across blocks, (**C**) α-FC slopes of dorsal V3 for during rest for responders, non-responders, and control participants across four sessions.

*Offline analyses of neurofeedback sessions*, based on artifact‑rejected data and spatial filters computed from the actual data, provided a complementary and more robust assessment of participants’ ability to modulate global α‑band FC of the target regions. In dorsal V3, the primary regions of interest previously identified as correlate of visual perception performance (Allaman et al. 2020), the comparison between the groups during neurofeedback was not significant, *t*(26) = 1.44, *p* = 0.162, 95% CI [–0.008, ∞), d = 0.54,see Fig. 4A). When restricting the analysis to left and right dorsal V3 - a significant group difference was observed only for the right dorsal V3, with the active group showing a greater increase compared to the control group (t_(26)_ = 2.50, *p* = 0.028, 95% CI [0.031, ∞), d = 0.95; see Fig. 3B). No difference was found in the left dorsal V3 (t_(26)_ = 0.57, *p* = 0.864; 95% CI [-0.050, ∞), d = 0.21 see Fig. 3A). No significant group difference was observed in the control region (left or right superior and middle frontal gyri) during neurofeedback training (t_(26)_ = -0.13, *p* = 1.000, 95% CI (-∞, 0.039], d=-0.05; see Fig. 3C).

A surface rendering of the voxel-wise statistical map further illustrates that group differences in global α-band connectivity modulation took place selectively around the targeted posterior brain areas and that they were most pronounced in the right hemisphere (see Fig. 3D).

The slope across *resting-state* recordings in dorsal V3 differed significantly between groups, with the Active group showing higher values than the Control group (t(26) = 2.25, *p* = 0.033, one-tailed; 95% CI [0.015, ∞); Cohen’s d = 0.85). The slope of global α-band FC of the right dorsal V3 showed a significant difference between groups (t_(26)_ = 2.42,  *p* = 0.034, 95% CI [0.030, ∞), d = 0.91; see Fig. 3F), whereas the left dorsal V3 did not differ significantly (t_(26)_ = 1.49, *p* = 0.221, 95% CI [-0.007, ∞), d = 0.56; see Fig. 3E). No significant difference was observed in the control region (t_(26_) = -1.89,  *p* = 0.105, 95% CI (-∞, -0.004], d=-0.71).In addition, the control group itself showed a significant increase relative to zero (t_(13)_ = 2.97, *p* = 0.016, one-tailed, 95% CI [0.019, ∞), d = 0.79). Again, the voxel-wise map demonstrates that effects were most pronounced around the target region in the right hemisphere (see Fig. 3H).

During both neurofeedback and rest, the control group exhibited a significant decrease in global α-band FC with right V3 (see Fig. 3B and F). Specifically, slopes were significantly below zero during neurofeedback (t(13) = − 3.20, *p* = 0.010, d = − 0.86) and at rest (t(13) = − 3.04, *p* = 0.016, d = − 0.81).

Group level α-band FC values for each neurofeedback training block are shown for both groups across Days 2 and 3 in the supplementary results B (Fig. S4).

Across the three analyses (NFB online: 32-time blocks; NFB offline: 6; Rest: 6), linear model fits were generally modest but adequate for estimating subject-level slopes. For NFB online, in dorsal V3d the active group showed a median R² of 0.27 [IQR = 0.33], while the control group had 0.09 [0.39]. For NFB offline, the active group had slope medians ranging from 0.18 to 0.26 [0.26–0.33] in V3d and 0.21 [0.28] in the control ROI, compared with 0.12–0.15 [0.40–0.53] in V3d and 0.38 [0.48] in the control group. During rest, the active group showed slope medians of 0.08–0.13 [0.16–0.25] in V3d and 0.14 [0.22] in the control ROI, whereas the control group showed 0.15–0.42 [0.39–0.54] in V3d and 0.11 [0.39] in the control ROI.

### Responder Analysis

In the active group, individual slopes of α-band FC in dorsal V3 during neurofeedback showed that seven participants exceeded the responder’s threshold (Fig. 4A). These participants were classified as *responders*, and the remaining seven as *non-responders*. The FC values for the three groups across the blocks are shown in Supplementary B, see Fig. S5.

*During neurofeedback* (see Fig. 4B), there was a significant difference in slope values in dorsal V3 among responders, non-responders, and controls (Kruskal–Wallis: χ² = 14.34, *p* < 0.001). Post-hoc pairwise comparisons (Bonferroni-corrected) revealed that responders had higher slopes than both non-responders (*p* = 0.001) and controls (*p* = 0.004). No significant difference was observed between non-responders and controls (*p* = 1.000).

*During rest*, there was a significant difference in slope values among responders, non-responders, and controls (Kruskal–Wallis: χ² = 7.62, *p* = 0.022). Responders had significantly higher slope values than controls (*p* = 0.019), while the difference between responders and non-responders (*p* = 0.581) and between non-responders and controls (*p* = 0.647) was not significant (see Fig. 4C).

To summarize, when comparing the active and control groups in dorsal V3 during both neurofeedback and rest, the active group maintained higher functional connectivity (FC) values, with the effect being more pronounced in the right hemisphere, whereas FC decreased in the control group. Within the active group, responders showed an increase in FC, while non-responders showed a decrease, similar to the control group, in both hemispheres.

**Fig. 5:**
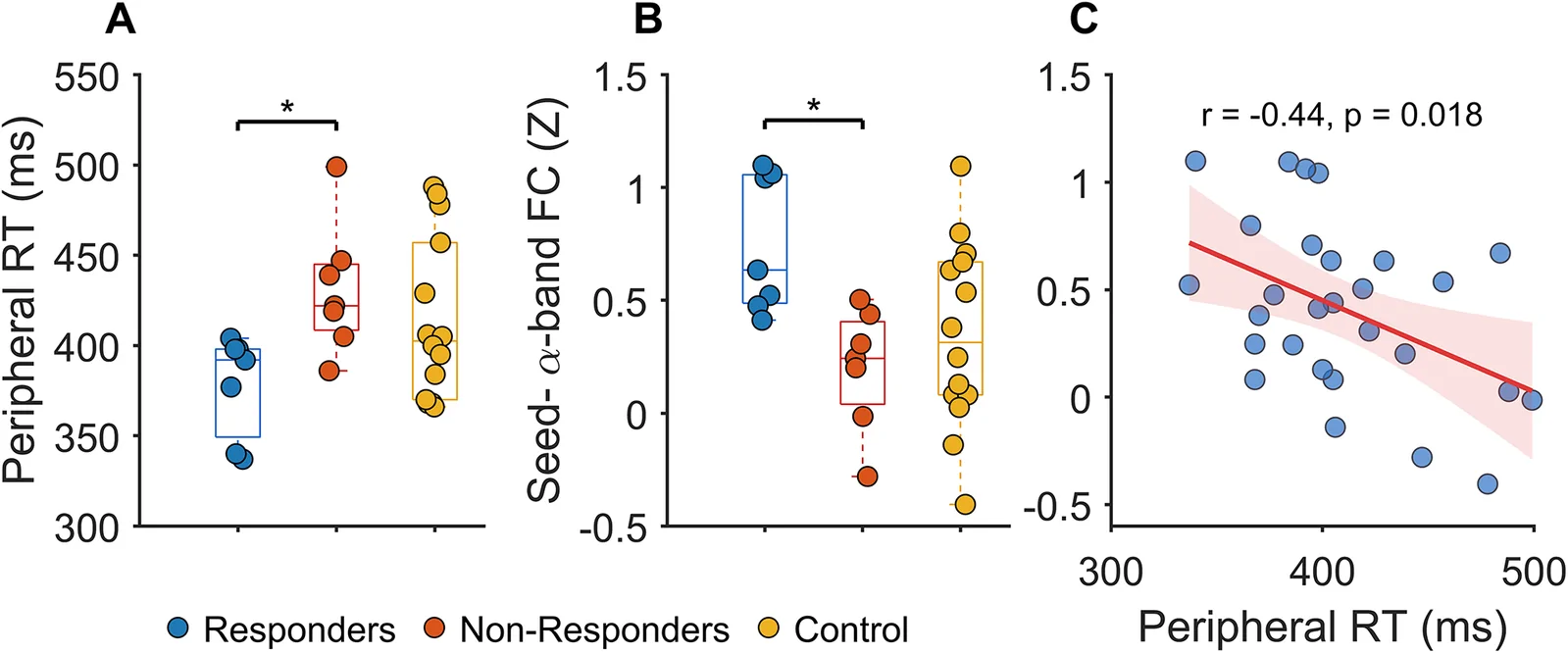
Baseline characteristics in responders, non-responders and controls. (**A**) Peripheral RT (ms) in the visual attention task at Day 1, (**B**) Seed-based FC between dorsal V3 and IPS at baseline. (**C**) Correlation between baseline V3–IPS α-band FC and peripheral RT (Day 1). The red line indicates the linear regression fit, and the shaded area represents the 95% CI

At baseline, the three groups did not differ in age (Kruskal–Wallis: χ² = 0.57, *p* = 0.753), sex (χ² = 1.29p = 0.524), corrected vision (χ² = 3.50, *p* = 0.174), handedness (χ² = 1.12, *p* = 0.571), meditation practice (χ² = 2.84, *p* = 0.241), contrast threshold (Kruskal–Wallis, χ² = 0.14, *p* = 0.931), or baseline resting α-band FC between dorsal V3 and the rest of the brain (Kruskal–Wallis χ² = 1.54p = 0.463). The only baseline difference between the three groups was in peripheral reaction times (Kruskal–Wallis, χ² = 6.41, *p* = 0.041); responders were faster (M = 378 ms) than non-responders (M = 431 ms; *p* = 0.035), while neither group differed significantly from controls (M = 414 ms), see Fig. 5A. In addition to behavioral differences, we found neurophysiological differences: the seed-based analysis between dorsal V3 and the IPS revealed a group effect (Kruskal–Wallis, χ² = 7.00, *p* = 0.030), with a significant difference between responders and non-responders (*p* = 0.031, see Fig. 5B). Moreover, stronger α-band FC between dorsal V3 and IPS was associated with faster peripheral reaction times (*r* = − 0.44, *p* = 0.018, see Fig. 5C). Thus, responders were characterized by greater neural and behavioral indices of visual attention at baseline.

### Behavioral Effects

**Fig. 6:**
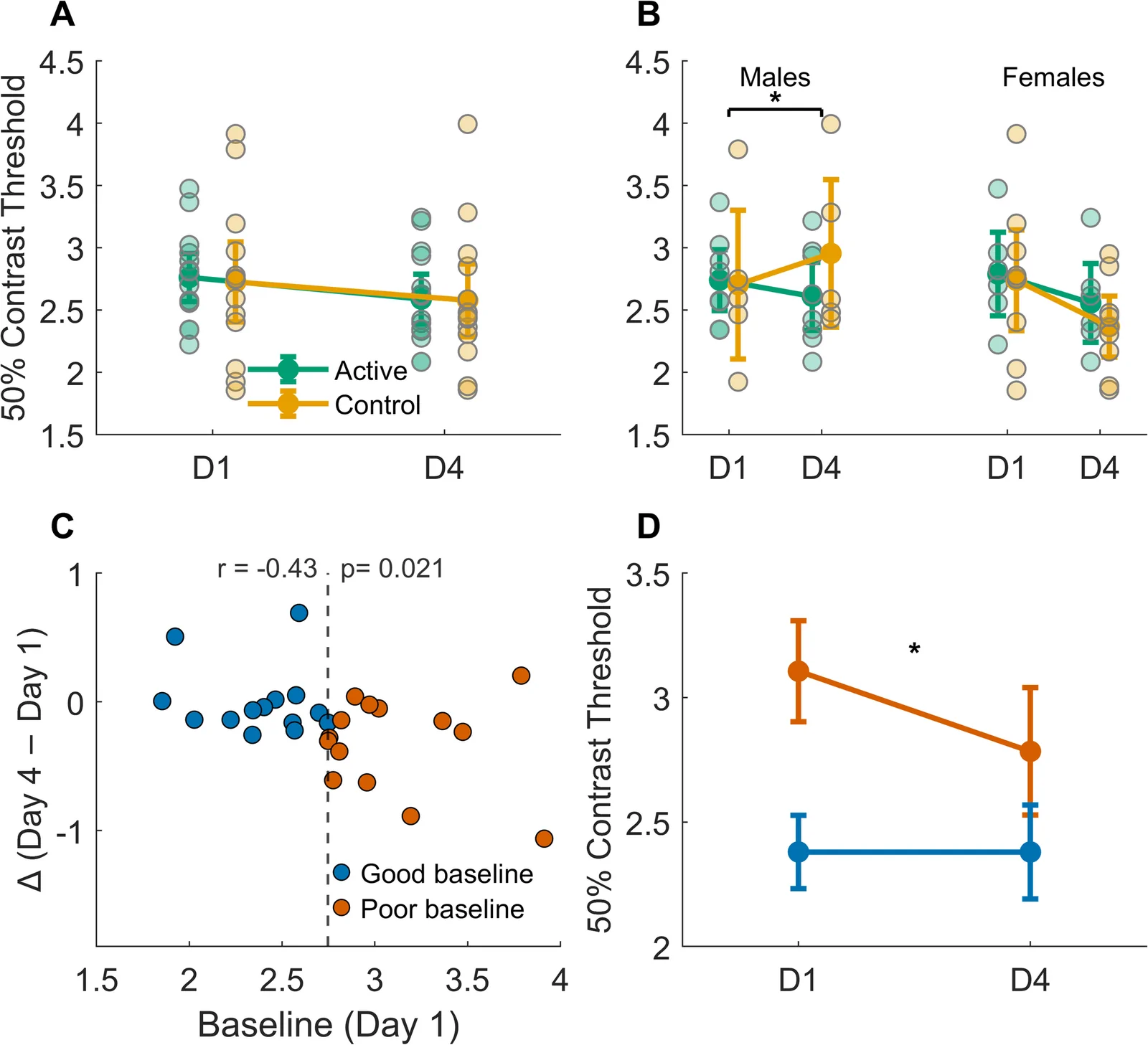
Visual detection task performance. (**A**) Group means (± 95% CI) and individual data points for 50% contrast threshold on Day 1 (D1) and Day 4 (D4) in the active (green) and control groups (orange). (**B**) Separation by sex (males, females) to illustrate the group × sex interaction. (**C**) Scatterplot showing the correlation between baseline threshold (Day 1) and the change in threshold (Day 4 – Day 1); the vertical dotted line marks the median split used for classification. (**D**) Participants stratified by baseline performance: those with poor baseline (higher thresholds, orange) showed greater improvement compared to those with good baseline (blue). Error bars indicate 95% CI

We next assessed behavioral effects of neurofeedback on the contrast detection threshold (Fig. 6A). There was a significant main effect of time (*F*_(1,26)_ = 5.62, *p* = 0.026, η²ₚ = 0.18), indicating that thresholds decreased from Day 1 to Day 4 across both groups. However, there was no significant main effect of group (F_(1,26)_ = 0.02, *p* = 0.897, η²ₚ = 0.001), nor was there a significant interaction (F_(1,26)_ = 0.03, *p* = 0.858, η²ₚ = 0.001). This suggests that both groups showed similar improvements over time, with no evidence for a differential effect of neurofeedback on behavioral performance.

In an exploratory analysis, including sex as a factor revealed a significant main effect of sex (F_(1,24)_ = 9.98, *p* = 0.004, η²ₚ = 0.29) and a significant interaction with group (F_(1,24)_ = 5.17, *p* = 0.032, η²ₚ = 0.17; see Fig. 6B). Men in the active group showed a greater reduction in contrast threshold compared with men in the control group (t_(11)_ = -2.75, *p* = 0.038, d=-1.57), whereas among women there was no difference between groups (t_(13)_ = 0.80, *p* = 0 0.881, d = 0.42).

Classifying participants as responders or non-responders did not reveal behavioral advantages for responders, as the overall change from pre- to post-training did not differ between the two groups (Mann–Whitney U, *p* = 0.620). Within groups, responders improved significantly from pre- to post-training (Wilcoxon, *p* = 0.016), whereas non-responders did not (Wilcoxon, *p* = 0.078). There was no significant correlation between the slope of FC change in dorsal V3 during neurofeedback and improvements in contrast threshold from Day 1 to Day 4 (*r* = 0.09, *p* = 0.653).

One possible reason for the lack of behavioral improvements despite increases in the neurophysiological target could be the presence of a ceiling effect. Baseline contrast threshold on Day 1 was negatively correlated with improvement (*r* = -0.43, *p* = 0 0.021), indicating that participants with poorer baseline performance showed greater gains, independent of group attribution (see Fig. 6C). There were significant effects of group (F_(1,26)_ = 18.02, *p* < 0.001, η²ₚ =0.41 ), time ( F_(1,26)_ = 7.14, *p* = 0.013,η²ₚ = 0.22), and their interaction (F_(1,26)_ = 7.13, *p* = 0.013,η²ₚ = 0.21). Participants with poor baseline performance improved significantly from Day 1 to Day 4 (t_(13)_ = 3.34, *p* = 0.005, see Fig. 6D), whereas those with good baseline performance did not improve (t_(13)_ = 0.011, *p* = 1.000).

Peripheral reaction time in the TAP-M Active Visual Field test improved significantly from Day 1 to Day 4 across all participants (F_(1,26)_ = 30.05, *p* < 0.001, η²ₚ = 0.54)), indicating overall faster responses. There was no significant main effect of group (F_(1,26)_ = 0.50, *p* = 0.486, η²ₚ = 0.02) and no group × time interaction (F_(1,26)_ = 0.12, pIMP = 0.727, η²ₚ = 0.005), suggesting that both the active and control groups improved similarly over time. Considering sex also revealed no significant effects of sex (F_(1,24)=_1.09, *p* = 0.306, η²ₚ = 0.01), or their interaction (F_(1,24)=_0.25, *p* = 0.624,η²ₚ = 0.01) on peripheral reaction time.

## Discussion

This paper presents a novel method for voluntary modulation of visual network interactions using auditory neurofeedback. By targeting α-band functional connectivity between visual areas and the rest of the brain, our approach focuses on large-scale neural communication previously linked to visual performance. Based on our pilot experiment, auditory feedback was selected because passive auditory stimulation does not engage visual areas or attention networks, ensuring that observed connectivity changes reflect learned control rather than the feedback modality itself.

Our results show that a subset of participants in the active group learned to upregulate connectivity in the visual area, whereas in the control group, it decreased.

The online neurofeedback signal reflected the participants’ real-time training experience, with increases in connectivity causing the feedback tone to rise and providing immediate reinforcement. Across the two training days, the active group, but not the control group, showed increased global α-band connectivity of the targeted regions. While these online measures are valuable for capturing real-time dynamics and support neurofeedback learning, they are nevertheless limited by the real-time processing constraints described above.

In contrast, our offline NFB FC analysis—based on rigorously pre-processed neurofeedback data and FC estimates derived from larger time blocks containing more epochs—offered more robust insights into the evolution of network interactions. At the group level, participants in the active condition maintained high global α-band FC of the targeted visual area, whereas the control group showed a decline. ROI analysis revealed the most sustained FC increase in the right dorsal V3, with complementary voxel-wise analysis showing additional increases in the right parietal and occipital cortices. The stronger effects in these right hemispheric areas may reflect strategies participants used to modulate the feedback signal. Their involvement in visuospatial attention and integration, as well as their proposed role in top-down modulation of visual cortex activity, may make them particularly responsive to the demands of the neurofeedback task (Bressler et al. 2008; Corbetta and Shulman 2002).

Moreover, responders to our neurofeedback intervention were characterized by higher α-band FC between the dorsal V3 and intraparietal sulcus, regions closely linked to visual attention. We speculate that these baseline connectivity differences may have given responders an advantage in learning to upregulate visual α-band FC with the rest of the brain, thereby supporting more efficient learning during training. The importance of parietal-to-visual modulation has also been observed in previous neurofeedback studies (Scharnowski et al. 2012, 2014).

The proportion of responders in our study was 50%, aligning with rates typically reported in neurofeedback research and closely matching the 48% observed in studies specifically targeting the visual cortex (Scharnowski et al. 2012). Not all individuals are able to effectively regulate neurofeedback or other brain–computer interface signals, particularly during the early stages of training. Regulation success likely depends on multiple factors, including the specific neural feature targeted, the amount of training, and individual differences (Alkoby et al. 2018).

Unexpectedly, global α-band functional connectivity of the visual regions showed a decrease across NFB training blocks in control-group participants, who did not train these regions, as well as in non-responders. In these groups, the observed pattern may reflect reduced attentional engagement or boredom due to difficulties in modulating the neurofeedback signal (Alkoby et al. 2018), with fatigue as a further contributing factor. Additionally, changes in impedance or noise over time cannot be ruled out (Luck 2014).

The effects observed in the dorsal V3 during neurofeedback persisted into the resting-state recordings obtained up to one day after training. The slope analysis revealed a positive upward trend in connectivity for responders compared to controls across the six resting state points. Given that training was limited to two sessions of 24 min each, these results demonstrate that even brief neurofeedback can enhance network communication. The change developed over sessions, peaking in the resting-state immediately after the second training day, likely reflecting short-term plastic changes in the visual network, as has also been reported previously following brief neurofeedback interventions (Ros et al. 2013). The control group showed a similar trend in the region they were training, but it did not reach statistical significance. This may indicate that different brain regions respond differently to neurofeedback training.

Behavioral improvements were observed primarily in male participants, suggesting a potential sex-related modulation of training effects. Notably, the impact on performance was shaped by multiple factors, with both sex and baseline ceiling effects likely influencing the observed results. The limited translation to behavioral effects may be due to several factors. First, the neural changes induced by a very short neurofeedback intervention may not have been large or sustained enough to produce detectable behavioral. Second, ceiling effects likely played a role, as participants with higher baseline performance had limited room for improvement, while those with poorer initial abilities showed greater behavioral gains. This highlights the importance of adjusting task difficulty, such as contrast level, for each participant, as has been done in previous studies (Allaman et al. 2020). Additionally, sex differences were evident, with males appearing to benefit more from neurofeedback than females. Among males, only those in the active group improved over time, whereas the control group showed a decline in performance. In contrast, females improved in both the active and control groups, which may reflect differences in attentional engagement or strategy use that differentially affect neurofeedback outcomes between sexes. However, the limited sample size precluded a separate analysis of males within responders, so these observations should be interpreted cautiously.

This study has several strengths, including the comparison of two feedback modalities (auditory and tactile, Supplementary Material A) to identify the most suitable approach, and the use of an active control group that followed the same protocol and instructions as the active group but trained on a brain region not directly associated with visual perception. This design helped ensure that observed effects were due to training the visual network, rather than general factors such as task engagement or expectancy. Randomized assignment to groups and a single-blind design further minimized selection and experimenter bias.

Limitations should be acknowledged. The intervention may have been too short for more robust effects. Individual MRI scans were not used for anatomical localization, which may have reduced spatial specificity. The relatively low number of EEG channels could have limited spatial resolution in FC estimates. Finally, using a fixed contrast level for the visual task across all participants may have contributed to ceiling effects in high performers.

Future work should translate this network-based neurofeedback approach to clinical populations to restore disrupted α-band interactions in individuals with acquired visual field deficits (Allaman et al. 2021). Unlike healthy individuals, these patients have substantial potential for improvement in visual performance, which may be realized through targeted modulation of functional connectivity. Enhancing the neurofeedback intervention through personalization, using each patient’s actual MRI for precise affected ROI localization, and providing intensified long-term training may enable more effective neurorehabilitation in this population.

## Supplementary Information

Below is the link to the electronic supplementary material.

Supplementary Material 1

Supplementary Material 2

## Data Availability

Data will be made available upon request.

## References

[CR1] Alkoby O, Abu-Rmileh A, Shriki O, Todder D (2018) Can we predict who will respond to neurofeedback? A review of the inefficacy problem and existing predictors for successful EEG neurofeedback learning. Neuroscience 378:155–164. 10.1016/j.neuroscience.2016.12.050 28069531

[CR3] Allaman L, Mottaz A, Kleinschmidt A, Guggisberg AG (2020) Spontaneous network coupling enables efficient task performance without local task-Induced activations. J Neurosci 40(50):9663–9675. 10.1523/JNEUROSCI.1166-20.2020 PMC7726528 33158966

[CR2] Allaman L, Mottaz A, Guggisberg AG (2021) Disrupted resting-state EEG alpha-band interactions as a novel marker for the severity of visual field deficits after brain lesion. Clin Neurophysiol 132(9):2101–2109. 10.1016/j.clinph.2021.05.029 34284245

[CR4] Amunts K, Mohlberg H, Bludau S, Zilles K (2020) Julich-Brain: A 3D probabilistic atlas of the human brain’s cytoarchitecture. Science 369(6506):988–992. 10.1126/science.abb4588 32732281

[CR5] Beffara B, Hadj-Bouziane F, Hamed SB, Boehler CN, Chelazzi L, Santandrea E, Macaluso E (2022) Dynamic causal interactions between occipital and parietal cortex explain how endogenous Spatial attention and stimulus-driven salience jointly shape the distribution of processing priorities in 2D visual space. NeuroImage 255:119206. 10.1016/j.neuroimage.2022.119206 35427770

[CR6] Bressler SL, Tang W, Sylvester CM, Shulman GL, Corbetta M (2008) Top-Down control of human visual cortex by frontal and parietal cortex in anticipatory visual Spatial attention. J Neurosci 28(40):10056–10061. 10.1523/JNEUROSCI.1776-08.2008 PMC2583122 18829963

[CR7] Busch NA, Dubois J, VanRullen R (2009) The phase of ongoing EEG oscillations predicts visual perception. J Neurosci 29(24):7869–7876. 10.1523/JNEUROSCI.0113-09.2009 PMC6665641 19535598

[CR8] Chapeton JI, Haque R, Wittig JH, Inati SK, Zaghloul KA (2019) Large-Scale communication in the human brain is rhythmically modulated through alpha coherence. Curr Biology: CB 29(17):2801–2811e5. 10.1016/j.cub.2019.07.014 PMC6736747 31422882

[CR9] Choi H-J, Zilles K, Mohlberg H, Schleicher A, Fink GR, Armstrong E, Amunts K (2006) Cytoarchitectonic identification and probabilistic mapping of two distinct areas within the anterior ventral bank of the human intraparietal sulcus. J Comp Neurol 495(1):53–69. 10.1002/cne.20849 PMC3429851 16432904

[CR10] Corbetta M, Shulman GL (2002) Control of goal-directed and stimulus-driven attention in the brain. Nat Rev Neurosci 3(3):201–215. 10.1038/nrn755 11994752

[CR11] Dalal SS, Zumer JM, Guggisberg AG, Trumpis M, Wong DDE, Sekihara K, Nagarajan SS (2011) MEG/EEG source Reconstruction, statistical Evaluation, and visualization with NUTMEG. Comput Intell Neurosci 2011(1):758973. 10.1155/2011/758973 PMC3061455 21437174

[CR12] de Haan GA, Heutink J, Melis-Dankers BJM, Brouwer WH, Tucha O (2015) Difficulties in daily life reported by patients with homonymous visual field defects. J Neuro-Ophthalmology: Official J North Am Neuro-Ophthalmology Soc 35(3):259–264. 10.1097/WNO.0000000000000244 25815856

[CR14] Dubovik S, Pignat J-M, Ptak R, Aboulafia T, Allet L, Gillabert N, Magnin C, Albert F, Momjian-Mayor I, Nahum L, Lascano AM, Michel CM, Schnider A, Guggisberg AG (2012) The behavioral significance of coherent resting-state oscillations after stroke. NeuroImage 61(1):249–257. 10.1016/j.neuroimage.2012.03.024 22440653

[CR15] Dubovik S, Ptak R, Aboulafia T, Magnin C, Gillabert N, Allet L, Pignat J-M, Schnider A, Guggisberg AG (2013) EEG alpha band synchrony predicts cognitive and motor performance in patients with ischemic stroke. Behav Neurol 26(3):187. 10.3233/BEN-2012-129007 PMC5214220 22713421

[CR16] Eickhoff SB, Stephan KE, Mohlberg H, Grefkes C, Fink GR, Amunts K, Zilles K (2005) A new SPM toolbox for combining probabilistic cytoarchitectonic maps and functional imaging data. NeuroImage 25(4):1325–1335. 10.1016/j.neuroimage.2004.12.034 15850749

[CR17] Garrison KA, Scheinost D, Worhunsky PD, Elwafi HM, Thornhill TA, Thompson E, Saron C, Desbordes G, Kober H, Hampson M, Gray JR, Constable RT, Papademetris X, Brewer JA (2013) Real-time fMRI links subjective experience with brain activity during focused attention. NeuroImage 81:110–118. 10.1016/j.neuroimage.2013.05.030 PMC3729617 23684866

[CR18] Gegenfurtner KR, Kiper DC, Levitt JB (1997) Functional properties of neurons in macaque area V3. J Neurophysiol 77(4):1906–1923. 10.1152/jn.1997.77.4.1906 9114244

[CR20] Guggisberg AG, Honma SM, Findlay AM, Dalal SS, Kirsch HE, Berger MS, Nagarajan SS (2008) Mapping functional connectivity in patients with brain lesions. Ann Neurol 63(2):193–203. 10.1002/ana.21224 PMC3646715 17894381

[CR19] Guggisberg AG, Dalal SS, Zumer JM, Wong DD, Dubovik S, Michel CM, Schnider A (2011) Localization of cortico-peripheral coherence with electroencephalography. NeuroImage 57(4):1348–1357. 10.1016/j.neuroimage.2011.05.076 21672634

[CR21] Hazelton C, Campbell P, Rowe FJ, Jonuscheit S, Kernohan A, Angilley J, Henderson CA, Langhorne P, Pollock A (2019) Interventions for visual field defects in people with stroke. Stroke 50(12):e419–e420. 10.1161/STROKEAHA.119.026516 PMC6532331 31120142

[CR22] Hepworth LR, Rowe FJ, Walker MF, Rockliffe J, Noonan C, Howard C, Currie J (2016) Post-stroke visual impairment: A systematic literature review of types and recovery of visual conditions. Ophthalmol Research: Int J 1–43. 10.9734/OR/2016/21767

[CR23] Iemi L, Chaumon M, Crouzet SM, Busch NA (2017) Spontaneous neural oscillations bias perception by modulating baseline excitability. J Neuroscience: Official J Soc Neurosci 37(4):807–819. 10.1523/JNEUROSCI.1432-16.2016 PMC6597018 28123017

[CR24] Iglewicz B, Hoaglin DC (1993) How to detect and handle outliers. ASQC Quality

[CR25] Kober SE, Wood G, Schuster S, Körner C (2024) Do miniature eye movements affect neurofeedback training performance? A combined EEG-Eye tracking study. Appl Psychophysiol Biofeedback 49(2):313–327. 10.1007/s10484-024-09625-6 PMC11101551 38492124

[CR26] Kothe C (2014) *Lab Streaming Layer (LSL)—A software framework for synchronizing a large array of data collection and stimulation devices.* https://github.com/sccn/labstreaminglayer

[CR27] Kujovic M, Zilles K, Malikovic A, Schleicher A, Mohlberg H, Rottschy C, Eickhoff SB, Amunts K (2013) Cytoarchitectonic mapping of the human dorsal extrastriate cortex. Brain Struct Function 218(1):157–172. 10.1007/s00429-012-0390-9 PMC3535362 22354469

[CR28] Larsson J, Heeger DJ (2006) Two retinotopic visual areas in human lateral occipital cortex. J Neurosci 26(51):13128–13142. 10.1523/JNEUROSCI.1657-06.2006 PMC1904390 17182764

[CR29] Luck SJ (2014) An introduction to the Event-related potential technique. MIT Press

[CR30] Manuel AL, Guggisberg AG, Thézé R, Turri F, Schnider A (2018) Resting-state connectivity predicts visuo-motor skill learning. NeuroImage 176:446–453. 10.1016/j.neuroimage.2018.05.003 29730496

[CR31] Mathewson KE, Gratton G, Fabiani M, Beck DM, Ro T (2009) To see or not to see: prestimulus α phase predicts visual awareness. J Neurosci 29(9):2725–2732. 10.1523/JNEUROSCI.3963-08.2009 PMC2724892 19261866

[CR34] Mottaz A, Solcà M, Magnin C, Corbet T, Schnider A, Guggisberg AG (2015) Neurofeedback training of alpha-band coherence enhances motor performance. Clin Neurophysiology: Official J Int Federation Clin Neurophysiol 126(9):1754–1760. 10.1016/j.clinph.2014.11.023 25540133

[CR32] Mottaz A, Corbet T, Doganci N, Magnin C, Nicolo P, Schnider A, Guggisberg AG (2018) Modulating functional connectivity after stroke with neurofeedback: effect on motor deficits in a controlled cross-over study. NeuroImage: Clin 20:336–346. 10.1016/j.nicl.2018.07.029 PMC6091229 30112275

[CR33] Mottaz A, Savic B, Allaman L, Guggisberg AG (2024) Neural correlates of motor learning: network communication versus local oscillations. Netw Neurosci 8(3):714–733. 10.1162/netn_a_00374 PMC11340994 39355447

[CR35] Newman MEJ (2004) Analysis of weighted networks. Phys Rev E 70(5):056131. 10.1103/PhysRevE.70.056131 15600716

[CR36] Nolte G, Bai O, Wheaton L, Mari Z, Vorbach S, Hallett M (2004) Identifying true brain interaction from EEG data using the imaginary part of coherency. Clin Neurophysiol 115(10):2292–2307. 10.1016/j.clinph.2004.04.029 15351371

[CR37] Palva S, Palva JM (2007) New vistas for α-frequency band oscillations. Trends Neurosci 30(4):150–158. 10.1016/j.tins.2007.02.001 17307258

[CR38] Ros T, Théberge J, Frewen PA, Kluetsch R, Densmore M, Calhoun VD, Lanius RA (2013) Mind over chatter: plastic up-regulation of the fMRI salience network directly after EEG neurofeedback. NeuroImage 65:324–335. 10.1016/j.neuroimage.2012.09.046 PMC5051955 23022326

[CR40] Rowe FJ, Wright D, Brand D, Jackson C, Harrison S, Maan T, Scott C, Vogwell L, Peel S, Akerman N, Dodridge C, Howard C, Shipman T, Sperring U, MacDiarmid S, Freeman C (2013) A Prospective Profile of Visual Field Loss following Stroke: Prevalence, Type, Rehabilitation, and Outcome. *BioMed Research International*, *2013*, 719096. 10.1155/2013/719096 PMC3782154 24089687

[CR39] Rowe FJ, Hepworth LR, Howard C, Hanna KL, Cheyne CP, Currie J (2019) High incidence and prevalence of visual problems after acute stroke: an epidemiology study with implications for service delivery. PLoS ONE 14(3):e0213035. 10.1371/journal.pone.0213035 PMC6402759 30840662

[CR41] Samaha J, Postle BR (2015) The speed of Alpha-Band oscillations predicts the Temporal resolution of visual perception. Curr Biology: CB 25(22):2985–2990. 10.1016/j.cub.2015.10.007 PMC4654641 26526370

[CR42] Scharnowski F, Hutton C, Josephs O, Weiskopf N, Rees G (2012) Improving visual perception through neurofeedback. J Neurosci 32(49):17830–17841. 10.1523/JNEUROSCI.6334-11.2012 PMC3520425 23223302

[CR43] Scharnowski F, Rosa MJ, Golestani N, Hutton C, Josephs O, Weiskopf N, Rees G (2014) Connectivity changes underlying neurofeedback training of visual cortex activity. PLoS ONE 9(3):e91090. 10.1371/journal.pone.0091090 PMC3946642 24609065

[CR44] Sekihara K, Nagarajan SS, Poeppel D, Marantz A (2004) Asymptotic SNR of scalar and vector Minimum-Variance beamformers for neuromagnetic source reconstruction. IEEE Trans Bio Med Eng 51(10):1726. 10.1109/TBME.2004.827926 PMC4041989 15490820

[CR45] Singh KD, Barnes GR, Hillebrand A (2003) Group imaging of task-related changes in cortical synchronisation using nonparametric permutation testing. NeuroImage 19(4):1589–1601. 10.1016/s1053-8119(03)00249-0 12948714

[CR46] Sitaram R, Ros T, Stoeckel L, Haller S, Scharnowski F, Lewis-Peacock J, Weiskopf N, Blefari ML, Rana M, Oblak E, Birbaumer N, Sulzer J (2017) Closed-loop brain training: the science of neurofeedback. Nat Rev Neurosci 18(2):86–100. 10.1038/nrn.2016.164 28003656

[CR47] Stam CJ, van Straaten ECW (2012) The organization of physiological brain networks. Clin Neurophysiology: Official J Int Federation Clin Neurophysiol 123(6):1067–1087. 10.1016/j.clinph.2012.01.011 22356937

[CR48] Stenroos M, Mäntynen V, Nenonen J (2007) A matlab library for solving quasi-static volume conduction problems using the boundary element method. Comput Methods Programs Biomed 88(3):256–263. 10.1016/j.cmpb.2007.09.004 18022274

[CR49] Thut G, Nietzel A, Brandt SA, Pascual-Leone A (2006) Alpha-band electroencephalographic activity over occipital cortex indexes visuospatial attention bias and predicts visual target detection. J Neuroscience: Official J Soc Neurosci 26(37):9494–9502. 10.1523/JNEUROSCI.0875-06.2006 PMC6674607 16971533

[CR50] Tzourio-Mazoyer N, Landeau B, Papathanassiou D, Crivello F, Etard O, Delcroix N, Mazoyer B, Joliot M (2002) Automated anatomical labeling of activations in SPM using a macroscopic anatomical parcellation of the MNI MRI single-subject brain. NeuroImage 15(1):273–289. 10.1006/nimg.2001.0978 11771995

[CR13] van Dijk H, Schoffelen J-M, Oostenveld R, Jensen O (2008) Prestimulus oscillatory activity in the alpha band predicts visual discrimination ability. J Neurosci 28(8):1816–1823. 10.1523/JNEUROSCI.1853-07.2008 PMC6671447 18287498

[CR51] Zimmermann P, Fimm B (2021) *Testbatterie zur Aufmerksamkeitsprüfung – Version Mobilität (TAP-M). Version 1.3.2. PSYTEST*. https://www.psytest.net/en/test-batteries/tap-m/purpose

[CR52] Xia, M., Wang, J., & He, Y. (2013). BrainNet Viewer: A Network Visualization Tool for Human Brain Connectomics. PLOS ONE, 8(7), e68910. 10.1371/journal.pone.0068910 PMC3701683 23861951

