# Supplementary material for: Towards Network-Based Neurofeedback of Visual Areas to Enhance Visual Perception
Source: Brain Topogr. 2026 Jan 22;39(2):18. doi: 10.1007/s10548-025-01172-5 (PMC13658376; doi:10.1007/s10548-025-01172-5)
Supplement: Supplementary file 1 [file 10548_2025_1172_MOESM1_ESM.docx]

Supplementary information A

Towards Network-Based Neurofeedback of Visual Areas to Enhance Visual Perception

*Despina Zlatkova^1,2^, Stephan M. Gerber^3^, Branislav Savic^2^, Tobias Nef^3,4^,
Adrian G. Guggisberg ^1,2,*^, Marija Ušćumlić^1,2^*

^1^	Division of Neurorehabilitation, Department of Clinical Neurosciences, University Hospital of Geneva, Av. de Beau-Séjour 26, Geneva 1211, Switzerland

^2^	Universitäre Neurorehabilitation, Universitätsklinik für Neurologie, Inselspital, University Hospital of Berne, Berne 3010, Switzerland

^3^	Gerontechnology and Rehabilitation Group, ARTORG Center for Biomedical Engineering Research, University of Bern, 3012 Bern, Switzerland

^4^	Department of Neurology, Inselspital, 3010 Bern, Switzerland

* Correspondence: [aguggis@gmail.com](mailto:aguggis@gmail.com)

**Pilot Experiment**

The pilot experiment evaluated and compared the neural effects of auditory and tactile stimulation on α-band FC in visual cortical areas. The goal was to identify the modality that did not induce confounding activation in or around the targeted region while supporting neurofeedback learning.

# 1. Materials and Methods

## 1.1 Participants

Six neurotypical participants (2 males; all right-handed; mean age = 24.3 years, SD = 1.36) completed both auditory and tactile feedback conditions of neurofeedback training. All participants volunteered without receiving monetary compensation.

## 1.2 Experimental design

Participants completed two neurofeedback sessions on separate days, with an average interval of 3.33 days between sessions (SD = 1.03). Each session started with a 7-minute eyes-closed resting-state EEG recording. This was followed by a 7-minute block of passive sensory stimulation, during which participants either listened to continuous auditory noise or received vibrotactile stimulation on the center of the inner palm. The sensory modality used during this block (auditory or vibrotactile) was also used for the subsequent neurofeedback training, which consisted of ten trials lasting 3 minutes each. The order of the conditions was counterbalanced across participants. Each session ended with a second 7-minute resting-state EEG recording. The procedure is illustrated in Figure 1.

## 1.3 Neurofeedback training

Participants underwent neurofeedback training to enhance α‑band functional connectivity between visual areas (V1, V2, V3d, and V3v), as delineated by the Julich Brain Atlas, and the rest of the brain. Feedback was delivered as white Gaussian noise or vibration, with intensity proportional to the α‑band FC strength between the target region and the rest of the brain. Each participant completed ten 3‑minute neurofeedback blocks. Instructions were the same as already described in Section 2.6, with additional methodological details provided in Sections 2.4-2.5.

**Figure S1. Experimental paradigm.** On each training day, participants completed resting-state EEG (7 min), EEG with auditory or tactile stimulation (7 min), a 30-min (10 x 3 min) auditory or tactile neurofeedback block, and a final resting-state EEG (7 min).

## 1.4 Offline analysis

Artifacts such as eye movements, muscle activity, and electrode noise were removed through visual inspection.

For both resting-state EEG and passive sensory stimulation, 300 clean epochs were selected for analysis. Details of the source-level functional connectivity (FC) calculation are provided in Section 2.5

To assess whether passive stimulation with the feedback modalities influenced FC, we compared voxel-wise FC between the passive stimulation and resting-state conditions. For each voxel, the FC value during rest was subtracted from the FC value during passive stimulation. This allowed us to identify brain regions where FC was modulated by sensory input. Our analysis focused on predefined regions of interest (ROI): the visual areas (V1, V2, V3d, V3v), the postcentral gyrus (involved in somatosensory processing), Heschl’s gyrus (auditory processing), and the intraparietal sulcus (IPS), a region implicated in visual attention.

We hypothesized that each feedback modality would induce changes in the corresponding sensory regions (e.g., auditory stimulation affecting the auditory cortex), but not in visual areas or regions associated with visual attention.

For the neurofeedback analysis, trials were grouped into five blocks, each corresponding to six minutes of training. Epochs were ranked based on the ratio of power in the α-band relative to surrounding frequencies, and the top 240 epochs were selected. To derive the spatial filter, we pooled all neurofeedback data from a given training day (i.e., all 5 blocks), and computed the filter on that combined set. This filter was then applied to each of the five neurofeedback blocks to estimate functional connectivity as described in section 2.5.

To assess neurofeedback effectiveness, we examined whether α-band functional FC within the target the dorsal V3) increased progressively over time. We hypothesized that both auditory and tactile neurofeedback would result in comparable increases in α-band FC in visual areas, indicating that both modalities are effective for neurofeedback training.

## 1.5 Statistical Analysis

To ensure appropriate test selection, data distributions were first assessed using the Shapiro–Wilk test for normality. As all data satisfied the assumptions for parametric testing (p > 0.05), paired t-tests were used throughout.

For passive stimulation, we tested (i) whether FC differed from rest, and (ii) whether the change differed between modalities (auditory vs. tactile).

For neurofeedback data, a linear regression was fitted across α-band FC in five consecutive neurofeedback blocks for each subject, and the slope of the regression line was extracted as an index of training-related change. These slope values were then compared against zero using one-sample t-tests and between modalities using paired t-tests.

# 2. Results

We first examined the effect of passive sensory stimulation on α-band FC. In the visual cortex (V1–V3, Fig. S2A), neither condition differed significantly from rest (audio: t_(5)_ = -0.55, p = 0.605; tactile: t_(5)_ = 0.24, p = 0.817) and no significant difference was observed between auditory and tactile stimulation ( t_(5)_ = -0.60, p = 0.575).

In the intraparietal sulcus (IPS, Fig. S2B), the tactile condition showed a significant FC decrease from rest (t_(5)_ = -3.51, p = 0.017), whereas the auditory condition did not ( t_(5)_ = -0.77, p = 0.477). Furthermore, tactile stimulation led to a significantly greater decrease in α-band FC compared to auditory stimulation (t_(5)_ = 2.58, p = 0.049).

For Heschl’s gyri (Fig. S2C), neither modality produced changes compared to rest (audio: t_(5)_ = 0.50, p = 0.639; tactile: t_(5)_ = -0.85, p = 0.435) and there were no significant differences between modalities ( t_(5)_ = 0.73,p = 0.497).

In the postcentral gyri (Fig. 2SD), FC decreased significantly from baseline in the tactile condition (t_(5)_ = -2.71, p = 0.042), but not in the auditory condition (t_(5)_ = -0.46, p = 0.663), although the difference between conditions was not significant (t_(5)_ = 1.02, p = 0.354).

Thus, auditory stimulation produced no changes of FC in our regions of interest, while tactile stimulation did. Auditory stimulation was therefore preferred for the main experiment.

**Figure S2: Effects of passive sensory stimulation on α-band functional connectivity (FC).** Changes in α-band FC (Z-scores) during passive auditory (blue) and tactile (orange) stimulation relative to rest. Each dot represents one participant; boxplots show group medians and interquartile ranges, with gray lines connecting within-subject data. Results are shown for (A) visual cortex (V1–V3), (B) intraparietal sulcus (IPS), (C) Heschl’s gyri, and (D) postcentral gyri.

We next evaluated neurofeedback effectiveness by analysing changes in α-band FC over time during training. Slope values were computed for each subject based on FC estimates across the five neurofeedback blocks. No significant difference in slope was found between the auditory and tactile groups over a single short feedback session (t_(5)_ = 0.56, p = 0.599).
