# Supplementary material for: Towards Network-Based Neurofeedback of Visual Areas to Enhance Visual Perception
Source: Brain Topogr. 2026 Jan 22;39(2):18. doi: 10.1007/s10548-025-01172-5 (PMC13658376; doi:10.1007/s10548-025-01172-5)
Supplement: Supplementary file 2 [file 10548_2025_1172_MOESM2_ESM.docx]

Supplementary information B

Towards Network-Based Neurofeedback of Visual Areas to Enhance Visual Perception

*Despina Zlatkova^1,2^, Stephan M. Gerber^3^ , Branislav Savic^2^, Tobias Nef^3,4^, Adrian G. Guggisberg ^1,2,*^, Marija Ušćumlić^1,2^*

^1^	Division of Neurorehabilitation, Department of Clinical Neurosciences, University Hospital of Geneva, Av. de Beau-Séjour 26, Geneva 1211, Switzerland

^2^	Universitäre Neurorehabilitation, Universitätsklinik für Neurologie, Inselspital, University Hospital of Berne, Berne 3010, Switzerland

^3^	Gerontechnology and Rehabilitation Group, ARTORG Center for Biomedical Engineering Research, University of Bern, 3012 Bern, Switzerland

Department of Neurology, Inselspital, 3010 Bern, Switzerland

**1. Results**

*1.1 Real-time FC*

To complement the slope analysis reported in the main manuscript, we provide a summary of the real-time α-band FC estimates that formed the basis for linear regression fits. Saved real-time FC values were first summarized for each participant and neurofeedback block using their median. Fig. S3 shows the group-level median and IQR of these values across participants for each block.

***Figure S3. Real-time α-band FC during neurofeedback training.*** *Data is shown for the active (****A****, green) and control (****B****, yellow) groups across two training days. Dots indicate group medians, and error bars represent the interquartile range (25th–75th percentile). Each group included* n *= 13 participants. The background shading codes for day 2 (blue) and day 3 (yellow).*

*1.2 Offline results*

Extended visualization of α-band FC across neurofeedback training and resting state recordings. This figure complements Figure 3 of the main manuscript by showing the spread of participant data.

***Figure S4. α-band FC across neurofeedback training and rest sessions.*** *Groups mean FC values (Z-scored; ±95% CI) are shown for the active (green) and control (orange) groups across different target regions and time points. Brain images illustrate the anatomical locations of each region of interest.* ***A- B****: Neurofeedback: α-FC time courses in left V3d (A), right V3d (B) across time blocks.* ***C–D****: Rest: α-FC time courses for left V3d (C), right V3d (D) across recording sessions. (****E–F****) Control regions (left or right superior and middle frontal gyri): changes across training blocks during neurofeedback (E) and rest recording sessions (F).*

***Figure S5. α-band FC across neurofeedback training and resting state recordings in responders, non-responders and control****. Median (± 25th–75th IQR) α-FC in bilateral dorsal V3 during neurofeedback training (****A****) and during rest (****B****).*

**2. Strategies for neurofeedback**

In the main experiment, we documented the cognitive strategies reported by participants during neurofeedback training. After each session, they provided short descriptions of their approaches, which were then categorized by the experimenter into four predefined types. The first category, breathing and relaxation, included strategies such as focusing on breathing, relaxation, or meditation. The second, imagery, referred to mental imagery, recalling memories. The third, cognitive, included strategies such as mental tasks, for example solving mathematical problems or recalling study material. Finally, the mixed category was used when participants combined two or more approaches. The percentage of each strategy category was calculated for day 2 and day 3 of training.

***Figure S6. Self-reported strategies during neurofeedback.*** *Strategy categories on day 2 (****A****) and day 3 (****B****). The strategies were categorized into four types: breathing/relaxation, imagery, cognitive, and mixed. Bars show the percentage of strategies reported within each category for responders (N=7), non-responders (N=7), and control participants (N=14).*
